# Secondary aorto-duodenal fistula successfully treated with a novel surgical method: A case report

**DOI:** 10.1016/j.ijscr.2020.05.041

**Published:** 2020-05-29

**Authors:** Takao Tsuneki, Yasuhiro Yuasa, Mizuki Fukuta, Hidenori Maki, Yuta Matsuo, Osamu Mori, Shohei Eto, Satoshi Fujiwara, Atsusi Tomibayashi, Takashi Otani

**Affiliations:** a Department of Surgery, Tokushima Red Cross Hospital, Japan; b Department of Endovascular treatment, Tokushima Red Cross Hospital, Japan

**Keywords:** Secondary aorto-duodenal fistula, Endovascular aneurysmal repair, Duodenectomy

## Abstract

**Introduction:**

Secondary aorto-duodenal fistula (sADF) is a complication of abdominal aorta artificial blood vessel replacement that often leads to death. However, an optimal operative method has not yet been established. We describe a patient who underwent artificial blood vessel reimplantation and duodenectomy after endovascular aneurysmal repair (EVAR) with positive outcomes.

**Presentation of case:**

An 84-year-old man underwent artificial blood vessel replacement in 2015. In September 2016, he visited our emergency department and was diagnosed with sADF based on computed tomography. Urgent EVAR was performed, followed by duodenal segmental resection on the next day without opening the syringeal part to minimize the pollution of the operative field. Artificial blood vessel reimplantation and omental flap transposition were performed. As of 2020, about 3 years after surgery, there has been no relapse of the infection.

**Discussion:**

Using our novel operative method, we can minimize exposure of the artificial blood vessel and surrounding tissue to intestinal juice and pus. We believe that this reduces the risk of postoperative artificial blood vessel reinfection.

**Conclusion:**

Controlling bleeding by EVAR and resection of the duodenum and artificial blood vessels as a group without opening the syringeal part can contribute to positive long-term results as this method minimizes the pollution of the duodenectomy surgical field.

## Highlights

• Our patient had previously undergone artificial blood vessel replacement.
• He was subsequently diagnosed with a secondary aorto-duodenal fistula.
• During duodenal segmental resection, the syringeal part was not opened.
• This avoided pollution of the operative field.
• There has been no relapse of infection for 3 years after surgery.

## 1 Introduction

Secondary aorto-duodenal fistula (sADF) after abdominal aorta artificial blood vessel replacement is rare (0.4–4%), but the complications that may result, including hemorrhage of the digestive tract and artificial blood vessel infection, can be fatal [1]. Surgery is the first choice for treatment; however, an optimal operative method has not been established. In this case report, we describe a patient who received artificial blood vessel reimplantation and duodenectomy after endovascular aneurysmal repair (EVAR) with positive outcomes. We also review the current literature.

This work has been reported in line with the SCARE criteria [2].

## 2 Presentation of case

Patient: 84 years, male, PS0

Chief complaint: Hematochezia

Medical history: Appendicitis, coronary artery disease

**Fig. 1:**
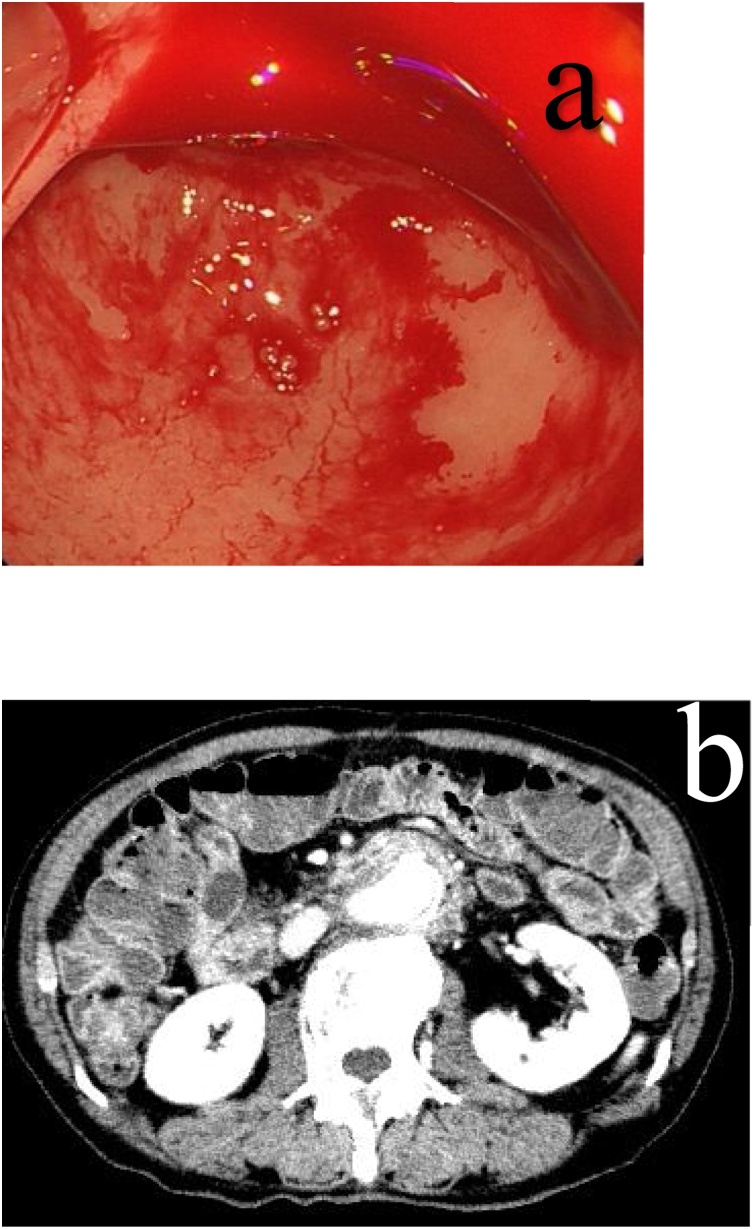
a. Endoscopy of the upper gastrointestinal tract shows the reflux of a large quantity of blood in the stomach from the duodenum. b. CT scan shows hyperplasia of the soft tissue and air bubbles around the artificial blood vessel.

**Fig. 2:**
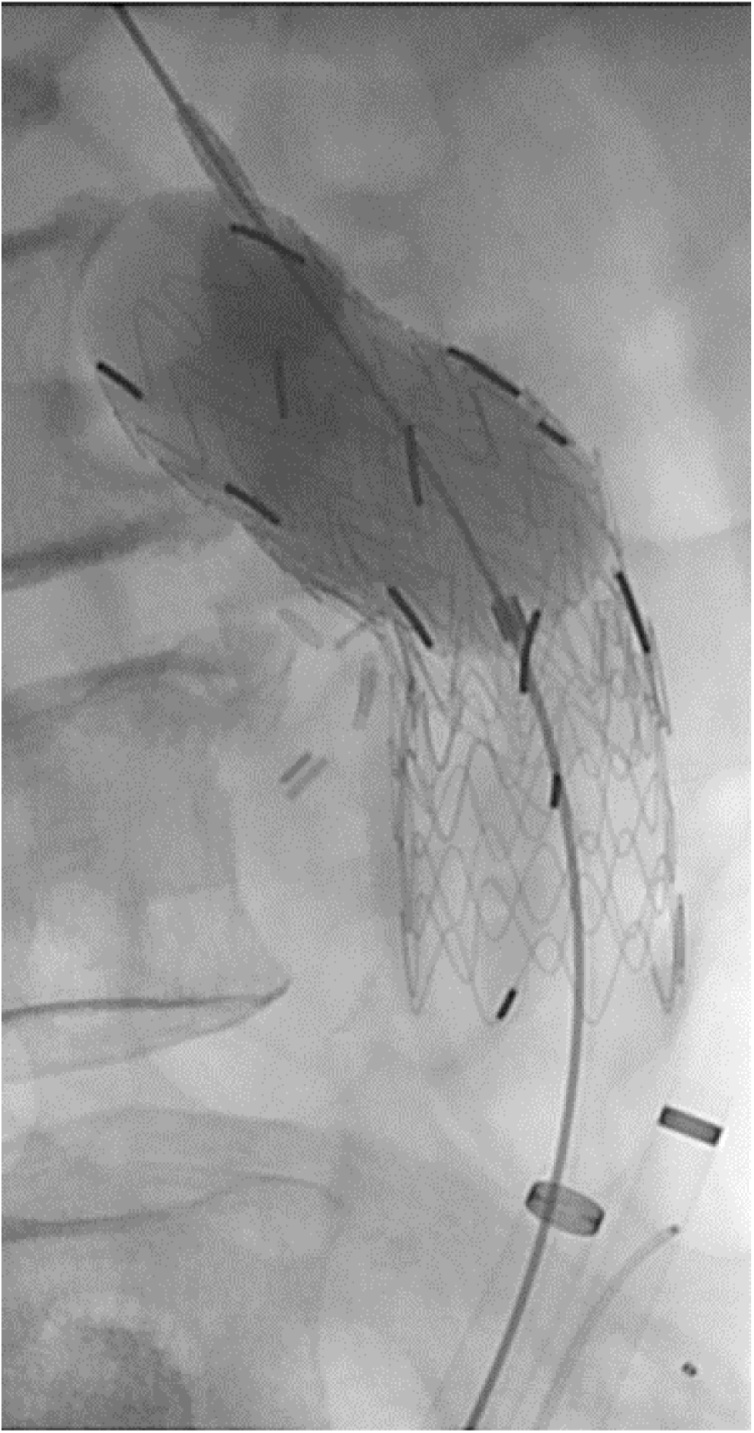
Because there is a leakage of the contrast media from the artificial blood vessel anastomotic region, a stent graft is inserted in the syringeal part.

**Fig. 3:**
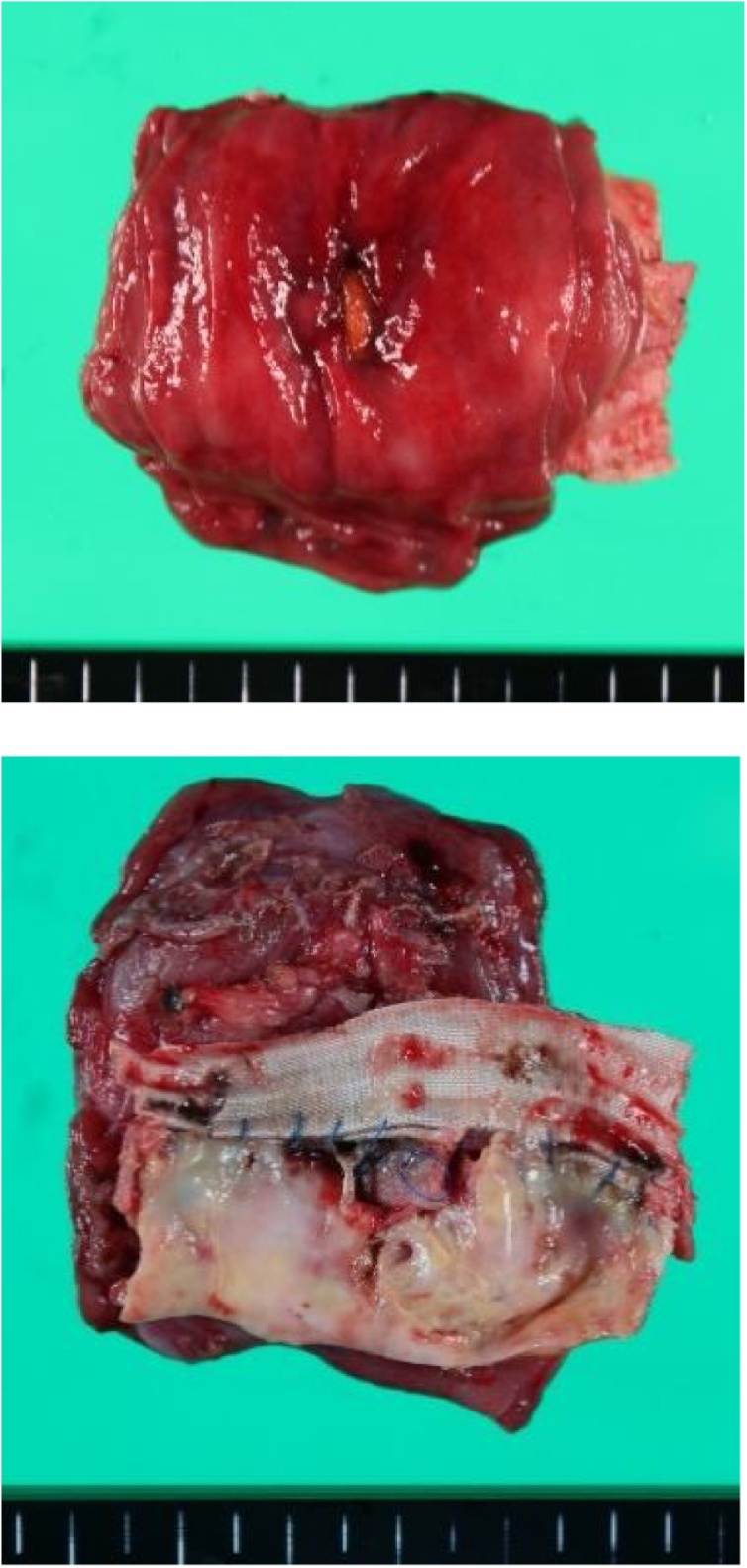
The aortic wall, artificial blood vessel, stent graft, and duodenum are stuck together in a group and resected.

**Fig. 4:**
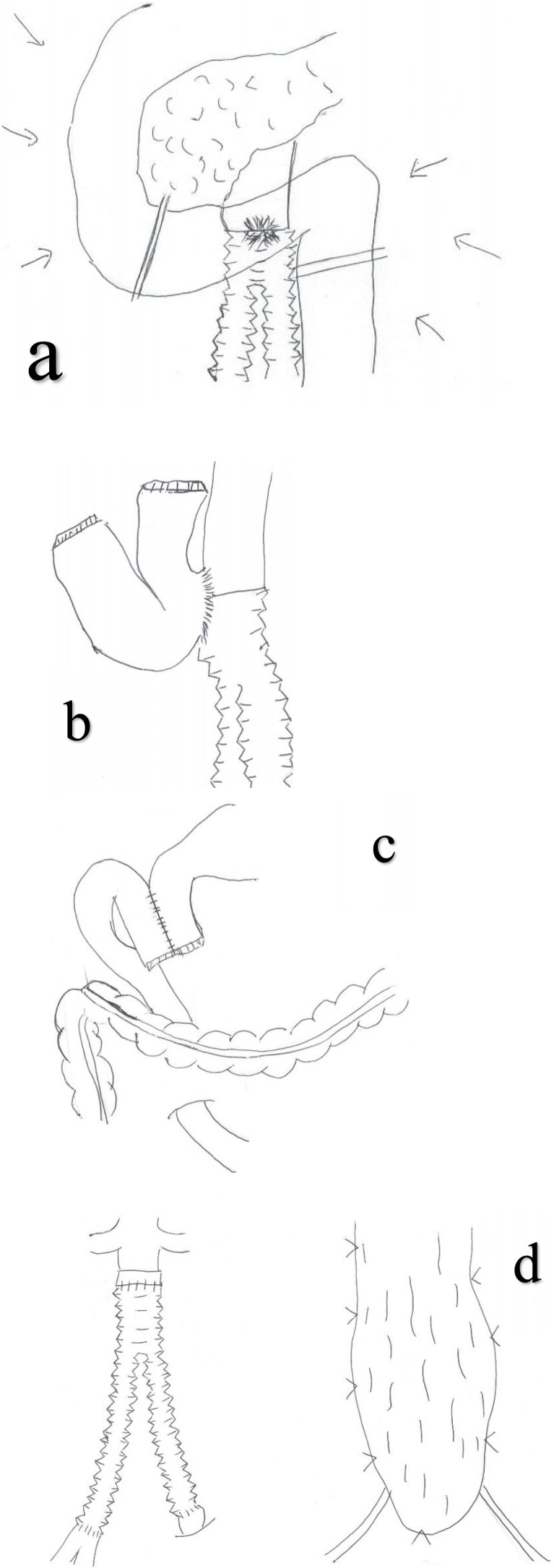
Surgical schema. a. Abrasion, mobilization of the duodenum, and separation of the actinal side and anal side are performed with automatic suture instruments. b. The syringeal part remains attached to the duodenum from the aortic wall without leaving it open. c. The side-to-side anastomosis of the duodenal descending limb and jejunum are performed via a posterior colon route. d. Artificial blood vessel reimplantation and omental flap transposition are performed.

Course: In January 2015, artificial blood vessel replacement was performed for abdominal aortic aneurysm, a right common iliac artery lump. Hematochezia was detected in September 2016 and the patient attended our emergency department. Mass bleeding was detected during endoscopy of the upper gastrointestinal tract and the examination was called off. sADF was diagnosed by computed tomography (CT), and urgent EVAR was performed. Given that hemostasis was obtained after EVAR and the patient’s overall status was stable, surgery for infection control was performed on the next day according to our hospital policies (Fig. 1, Fig. 2, Fig. 3, Fig. 4).

Blood test findings: Anemia and an increase of the inflammatory reaction (Hb, 7.5 g/dL; WBC, 12000/μL; CRP, 7.78 mg/dL) were detected.

Upper gastrointestinal tract endoscopy findings: Blood was absent in the stomach at endoscopic insertion. During attempted insertion into the duodenum, a large quantity of blood flowed backward into the stomach from the pylorus, and a reduction in blood pressure and a rise in heart rate were observed, prompting cessation of the examination.

CT scan findings: A large quantity of liquid effusion from the stomach to the jejunum was detected. Soft part levels and air bubbles around the artificial blood vessel were shown.

EVAR time findings: We found leakage of contrast media from the artificial blood vessel anastomotic region. We detained an aortic cuff (Gore Excluder Aortic cuff) of 23 mm × 33 mm at a syringeal position, repeated the same things, and added it to a central side as well as the peripheral side, respectively. We confirmed that there was no leakage of the contrast media.

Surgical method: Artificial blood vessel replacement, duodenal segmental resection, and omental flap transposition were performed.

Perioperative findings: The third and fourth parts of the duodenum strongly adhered to the aorta, which was evaluated with the fistula. We performed duodenal segmental resection without opening the syringeal part of the duodenum in an attempt to minimize exposure of the artificial blood vessel and surrounding tissue to intestinal juice and pus. Firstly, we performed the Kocher maneuver with automatic suture instruments in the middle of the third part of the duodenum. We separated the jejunum with an automatic anastomosis device and pushed forward the detachment on the actinal side. Resection of the intestinal tract was completed by attaching the abrasion to the aorta. We lifted the jejunum toward the transverse mesocolon at the right side and performed a duodenal and side-to-side anastomosis. We resected a portion of the aortic wall of the central side, artificial blood vessel, stent graft, and duodenum, which stuck together in a group, and performed artificial blood vessel reimplantation (Y graft) and omental flap transposition.

Postoperative course: Postoperative gastric tube drainage was needed before the start of dietary intake. Oral intake began from day 10 after surgery, and the patient was discharged from the hospital to the care of a nearby doctor for rehabilitation on day 35 after surgery. Antibiotics were withdrawn on day 39 after surgery. As of 2020, about 3 years after surgery, there has been no relapse of the infection.

## 3 Discussion

A primary aorto-duodenal fistula may develop due to aneurysm, infection, trauma, irradiation, and the like, and the sADF may develop after the replacement of a synthetic conduit. Postoperative sADF occurs in 0.4–4% of cases [1].

Infection and chronic mechanical irritation are considered possible mechanisms of the pathogenesis of sADF. The infection hypothesis involves the failure of the anastomotic region with an infection that forms a false aneurysm. It is believed that this causes intestinal inflammation and forms a fistula. On the other hand, the chronic mechanical irritation hypothesis involves the adherence of the intestinal tract to an artificial blood vessel anastomotic region, resulting in erosion in the intestinal tract by an aortic beat, and then penetration occurs. Infection finally occurs in the anastomotic region, following which the anastomotic region fails and then forms a fistula [3]. The latter hypothesis is considered relevant to our patient.

Hemorrhage of the digestive tract, pain, and pulsatile mass are the classic triad of symptoms, but only 0–40% of patients show these three symptoms. It is said that characteristic preceding bleeding, called “herald bleeding,” precedes fatal massive bleeding in approximately two out of three patients. The time from “herald bleeding” to massive bleeding is 6 h or more in 70%, more than 24 h in 50%, and 1 week or more in 29% of patients [4].

In terms of diagnosis, it is reported that only 13–38% of patients are diagnosed with upper gastrointestinal tract endoscopy alone because in sADF, 80% of fistulation sites are in the horizontal portion of the duodenum, and active bleeding cannot be confirmed at the time of preceding bleeding [5]. Many reports have found a CT scan to be useful for diagnosis. Findings such as the soft tissue shadow around the contrasted graft, free air around the graft, signs of liquid effusion, false lump formation, and the like are important on CT scan [6]. We were able to obtain a diagnosis from the CT scan in this case.

As the principal aims of treatment are the control and revascularization of bleeding, and the control of the infection, we believe it is important to use an operative method that divides the aorta and duodenum [7,8]. Surgery for complete removal of the old artificial blood vessel has been recommended after non-anatomical revascularization in the aorta. However, the mortality in the early postoperative period from complications such as aortic failure or graft obstruction, and the like is 23–44%, and this is not acceptable. In recent years, reports on the improvement of mortality and complication rates with anatomical revascularization have been published [4,9,10]. As a result, according to Batt et al., who compared an anatomical revascularization group with a non-anatomical revascularization group of sADF patients, non-anatomical revascularization was not superior to anatomical revascularization [11]. Hashimoto et al. reported positive outcomes of anatomical revascularization in Japan [9]. However, the overall status is often unstable due to bleeding at the sADF onset. In such cases, Eto et al. insisted that bleeding must be controlled first via EVAR, and after the patient is stabilized, “bridge therapy” should be performed as the radical cure with artificial blood vessel removal via abdominal surgery immediately following EVAR [12]. The overall status in our patient was poor due to massive bleeding at diagnosis, but we were able to accomplish surgery with a stable status on the next day after EVAR surgery. We also include 1) simple syringeal suture closure, 2) fistulectomy + suture closure, and 3) horizontal portion of duodenum resection + gastrointestinal tract reconstructive operative methods in the duodenum [13,14]. Currently, there is no consensus in terms of the optimal operative method despite reports that postoperative infection relapse and the recurrence of the aorta intestinal fistula may be fatal in patients with sADF [15]. Given this, perioperative infection control is considered to largely influence long-term results. To control infection, we performed duodenal segmental resection. During reconstruction, we lifted the jejunum to the transverse mesocolon at the right side and performed side-to-side anastomosis to ensure that the duodenal descending limb and jejunum did not pollute an artificial blood vessel, even if a ruptured suture occurred. Furthermore, to prevent pollution to the artificial blood vessel circumference by opening the syringeal part, we separated the actinal side and the anal side of the syringeal part with automatic suture instruments, and it was performed in a group for aortic valve replacement, and we performed resection without opening the syringeal part. As leakage of intestinal juice was minimized by separation with automatic suture instruments and opening of the syringeal part was not necessary, we were able to minimize the pollution. This operative method is assumed to be suitable for sADF. There is not much evidence about the optimal administration period of the antibiotic agents after surgery; however, our patient was cured approximately 6 weeks after surgery and there has been no subsequent relapse of the infection [[16], [17], [18]].

## 4 Conclusion

The main limitation to this case report was that it only describes one case, so this limits the generalizability of the results. This procedure needs to be performed in a number of subsequent cases to confirm the effectiveness of our novel procedure for sADF.

In patients with sADF, a vascular surgeon and digestive organ surgeon should cooperate in the diagnosis and the treatment. There is still room for improvement to achieve long-term results in terms of infection control and operative method. Further case reports are needed to gather more information regarding the treatment of sADF.

## Declaration of Competing Interest

We have no conflicts to declare on this literature.

## Sources of funding

There is no funding sources.

## Ethical approval

The ethical approval was given from our institution.

## Consent

Written informed consent was obtained from the patient for publication of this case report.

## Author contribution

Takao Tsuneki：Operating Surgeon and writing the paper.

Yasuhiro Yuasa：Assistant Surgeon, Supervisor and writing the paper.

Mizuki Fukuta：Writing the paper.

Hidenori Maki：Writing the paper.

Yuta Matsuo：Writing the paper.

Osamu Mori：Writing the paper.

Shohei Eto：Writing the paper.

Satoshi Fujiwara：Writing the paper.

Atsusi Tomibayashi：Writing the paper.

Takashi Otani：Assistant Surgeon and Writing the paper.

## Guarantor

Takao Tsuneki.

## Provenance and peer review

Not commissioned, externally peer-reviewed.
